# Effects of sleeve gastrectomy and Roux-en-Y gastric bypass on the pharmacokinetics of gabapentin and pregabalin: A cohort study

**DOI:** 10.1371/journal.pone.0319912

**Published:** 2025-03-26

**Authors:** Georgios Schoretsanitis, Hege-Merete Krabseth, Magnus Strømmen, Arne Helland, Olav Spigset

**Affiliations:** 1 The Zucker Hillside Hospital, Psychiatry Research, Northwell Health, Glen Oaks, New York, United States of America; 2 Department of Psychiatry, Donald and Barbara Zucker School of Medicine at Northwell/Hofstra, Hempstead, New York, United States of America; 3 Department of Psychiatry, Psychotherapy and Psychosomatics, Hospital of Psychiatry, University of Zurich, Zurich, Switzerland; 4 Department of Clinical Pharmacology, Clinic of Laboratory Medicine, St. Olav University Hospital, Trondheim, Norway; 5 Department of Clinical and Molecular Medicine, Norwegian University of Science and Technology, Trondheim, Norway; 6 Centre for Obesity Research, Clinic of Surgery, St. Olav University Hospital, Trondheim, Norway; National Healthcare Group, SINGAPORE

## Abstract

**Background:**

Bariatric surgery may affect the pharmacokinetics of medications by altering the gastrointestinal physiology. Pharmacokinetic changes of first-line neuropathic pain medications such as gabapentin and pregabalin following bariatric treatment have barely been investigated.

**Methods:**

In our prospective five-case study we included gabapentin- or pregabalin-treated patients undergoing bariatric surgery at hospitals in Central Norway. Concentrations of gabapentin and pregabalin were assessed using serial blood samples over a dose interval, preoperatively and one, six and twelve months postoperatively. The primary outcomes of the study included changes in area under the time-concentration curve (AUC) with secondary outcomes comprising full pharmacokinetic profiling. Formal statistical testing was not performed due to few cases.

**Results:**

Three pregabalin-treated obese patients undergoing Roux-en-Y gastric bypass (RYGB) and two gabapentin-treated patients undergoing RYGB (n = 1) and sleeve gastrectomy (SG) (n = 1) were included. Largest changes for dose-adjusted AUC values after surgery were seen in pregabalin-treated patients at one and six months (average increases of 53% one month and 28% 6 months postoperatively). In the patients on gabapentin, mean AUC changes were less than 10% from baseline throughout the study period. The inter-individual variation was high.

**Conclusion:**

Postoperative pharmacokinetic changes for gabapentin were minimal, but for pregabalin we observed more pronounced changes, particularly in one patient. Due to few cases, the results should be interpreted with caution. Given the large inter-individual variation, therapeutic drug monitoring could be considered to capture pharmacokinetic changes and guide dose adjustments postoperatively.

## Introduction

Bariatric surgery is a common management option for patients with obesity [1]. At the moment, the most frequently performed types of bariatric operations include the sleeve gastrectomy (SG) and the Roux-en-Y gastric bypass (RYGB) [2]. These surgeries may lead to physiological alterations which can impact gastric mixing, pH and emptying as well as gastrointestinal transit time, potentially affecting the absorption of medications [3]. Moreover, absorptive area reduction in the gastrointestinal tract can lead to alterations in bioavailability [4]; specifically, bypassing the duodenum as part of the RYGB may limit the metabolism of medications mediated by cytochrome P450 (CYP) enzymes in the wall of the proximal intestinal tract [4]. Further, the pharmacokinetic impact of the RYGB may be related to the total length of bowel preoperatively as well the length of bypassed bowel [5]. Additionally, the gastrojejunostomy size can also exert an important role due to its impact on gastric emptying time [6]. In the long term, lipophilic medications may redistribute as a result of adipose tissue mass decrease postoperatively [7]. Moreover, parallel to body weight decrease, a gradual increase of the activity of some CYP isoenzymes, particularly CYP2C19 and CYP3A4, may also take place [8,9]. Consequently, the complex interplay of the physiological effects of bariatric surgeries introduces a challenge in postoperative pharmacological treatment.

Gabapentin and pregabalin, also known as gabapentinoids, are first-line treatment options for neuropathic pain, but are regularly used as off-label medications for other forms of pain [10]. They have also been studied in the treatment of postoperative pain after bariatric surgery [11,12]. Over the past years there has been a substantial increase in prescriptions of gabapentinoids for a broad range of pain indications [13]. For instance, in the US, both medications were among the ten most commonly prescribed in 2016 [13].

So far, very little published data exists on the effects of bariatric surgery on the pharmacokinetics of gabapentinoids. This lack is even more striking considering the prevalence of chronic pain among patients with obesity and the prescription trends of gabapentinoids [14]. We are aware of one longitudinal case series assessing a sample of patients on various psychotropic medications mainly undergoing RYGB, with a median postoperative follow-up of 379 days [15]. This study described dose-adjusted through serum concentrations in two gabapentin- and seven pregabalin-treated patients. Although the pattern was mixed, the authors generally reported a concentration increase, which was strikingly large for pregabalin, during the preoperative low-calorie dieting phase. Postoperatively, concentrations gradually decreased, but were, at least for some patients on pregabalin, still above baseline at the end of the study period. Interestingly, in pregabalin-treated patients the increase in serum concentrations during the preoperative low-calorie dieting phase covaried with the degree of weight loss during the same period [15].

The aim of our study was to assess the impact of RYGB and SG on the pharmacokinetics of gabapentin and pregabalin measured at one, six and twelve months postoperatively, as compared to preoperative values.

## Methods

### Study design and study population

This study is part of a larger pharmacokinetic platform investigation, “Changes in oral health and pharmacokinetics of drugs after bariatric surgery (BAR-MEDS)”. The study methodology has been described in more detail previously [16]. In this case-only prospective study we investigated a cohort of gabapentin- or pregabalin-treated patients undergoing RYGB or SG between 2016 and 2021. Specifically, the study recruitment took place from November 2nd, 2016 to January 27th, 2021. Data was collected at the Centre for Obesity Research at St. Olav University Hospital, Trondheim, Norway. All patients underwent a multidisciplinary preoperative screening in the Obesity Clinic; further, patients were informed in advance about the potential influences of the bariatric surgery on the pharmacokinetics of their medications and were subsequently invited to participate in our study. The Regional Committee for Medical and Health Research Ethics in Mid Norway (ref. 2016/1145) approved the platform study, which is also registered at www.clinicaltrials.gov (NCT03476538). The study was performed in accordance with the ethical standards as laid down in the 1964 Declaration of Helsinki and its later amendments or comparable ethical standards. All patients provided written, informed consent.

For the pharmacokinetic testing, we acquired serial blood samples preoperatively and one, six and twelve months postoperatively. No specific diet regimens were applied at the days of pharmacokinetic assessments. Blood samples were obtained at 0, 0.5, 1, 1.5, 2, 2.5, 3, 3.5, 4, 6, and 12h after dose intake in patients taking the medication twice daily, whereas the 12h-sample was substituted with a sample obtained 8h after drug intake in patients taking the drug thrice daily. After centrifugation and pipetting, serum samples were stored at -80 °C until analysis.

Body composition was assessed using a multifrequency impedance analyzer (InBody 720, Seoul, South Korea).

### Drug quantification

Serum concentrations of gabapentin and pregabalin were assessed using an ultra-performance liquid chromatography–tandem mass spectrometry (UPLC–MSMS) method developed and validated in our laboratory. Samples underwent protein precipitation with ice cold acetonitrile with 1% formic acid and filtration through a phospholipid removal plate (Ostro^TM^, Waters; Milford, MA, USA) prior to analysis. Pregabalin-d_4_ and gabapentin-d_4_ were used as internal standards. The chromatographic system was an Acquity UPLC I-Class instrument equipped with a BEH phenyl 1.7 µm, 2.1 x 30 mm column (Waters) and a Van Guard BEH phenyl pre-column (1.7 µm, 2.1 x 5 mm). The mobile phase consisted of 2 mmol/L ammonium acetate and 0.1% formic acid in H_2_O (A) and in methanol (B), using gradient elution. The MS/MS detection was performed on a Xevo TQ-S (Waters; Manchester, UK) with positive electrospray ionization and multiple reaction monitoring (pregabalin: m/z 160.1 >  97.0 and m/z 160.1 >  142.0; gabapentin: m/z 172.1 >  137.0 and m/z 172.1 >  95.1). The calibration range was 1–200 µmol/L for both analytes. The inter-day coefficients of variation were in the range of 0.6–2.1% at the three concentrations tested.

Concentrations are reported in micromolar units (µmol/L). To convert to mass units (mg/L), multiply by 0.16 for pregabalin and by 0.17 for gabapentin.

### Pharmacokinetic analyses

When daily dose was changed in a patient during the study period, concentrations were adjusted to the dose the patient used the majority of study days. Subsequently, trough concentrations (C_0_; measured immediately before the morning dose intake), maximum concentrations (C_max_) and the times to achieve C_max_ (T_max_) were directly assessed using the dose-adjusted concentrations. The pharmacokinetic program package Kinetica, version 4.3 was used to assess other pharmacokinetic variables (Thermo Fisher Scientific; Waltham, MA, USA).

We calculated area under the time-concentration curve (AUC) during a dose interval (i.e., from 0 to 12 or 0 to 8 hours after dose intake for patients taking the drug twice or thrice daily, respectively), using a mixed log-linear model. To estimate apparent oral clearance (Cl/F) the dose ingested at t = 0 was divided by the AUC. Using a non-compartment model, the parameter describing the decrease of the log-concentration (the elimination constant; λ_z_) was estimated using the best-fit log-linear regression line of the samples representing the elimination phase. Elimination half-life (T_1/2_) was estimated as ln2/λ_z_ and the apparent volume of distribution (V_d_/F) was calculated as (Cl/F)/λ_z_.

### Outcomes

The primary outcome was the systemic exposure of gabapentin and pregabalin measured as AUC. Secondary outcomes included additional pharmacokinetic variables. The relative changes from baseline were used to quantify the post-operative changes in pharmacokinetics of medications and biometrics of patients. Given the small size of the cohort formal statistical testing was not feasible.

## Results

Two gabapentin- and three pregabalin-treated patients were included; four underwent RYGB and one SG. Baseline characteristics of the patients are available in Table 1. All five patients were Caucasians. Four patients (two treated with gabapentin and two with pregabalin) had a diagnosis of neuropathic pain (one gabapentin-treated patient also had a diagnosis of chronic back pain), whereas one pregabalin-treated patient had chronic back pain with no specific mention of neuropathic pain. Clinical biochemistry results are provided in S1 Table. Pharmacokinetic baseline testing for patient 1 took place 6 days into the preoperative low-calorie dieting phase, whereas for the other patients baseline testing took place before the start of the dieting phase.

**Table 1:** Patient characteristics of five subjects undergoing bariatric surgery during treatment with gabapentin (patients 1-2) or pregabalin (patients 3-5).

**Patient number**	**Age**	**Sex**	**Drug**	**Type** **of surgery**	**Daily dose**				**Other medications**
					**Before surgery**	**1 month after surgery**	**6 months after surgery**	**12 months after surgery**	
1	37	♀	Gabapentin	SG	600 mg t.d.s.	600 mg t.d.s.	600 mg t.d.s.	1200 mg t.d.s.^1^	Paracetamol^2^, pantoprazole,^3^
2	59	♀	Gabapentin	RYGB	300 mg t.d.s.	NA	300 mg t.d.s.	300 mg t.d.s.	Alendronate, metformin, omeprazole, atorvastatin, semaglutide, acetylsalicylic acid^3^
3	62	♀	Pregabalin	RYGB	150 mg t.d.s.	150 mg t.d.s.	150 mg t.d.s.	150 mg t.d.s.	Methotrexate, bumetanide, pantoprazole, levothyroxine, liothyronine, paracetamol^2^, naproxen/esomeprazole^4^, phenylpropanolamine^5^
4	49	♂	Pregabalin	RYGB	225 + 150 + 225 mg	225 + 150 + 225 mg	225 + 150 + 225 mg	NA	Pravastatin, pantoprazole, acetylsalicylic acid, levomepromazine^3^, lercanidipine^6^, ramipril^6^
5	44	♂	Pregabalin	RYGB	300 mg b.d.	300 mg b.d.	300 mg b.d.	300 mg b.d.	Esomeprazole^5^, paracetamol^6^, fluoxetine^6^, etoricoxib^6^

b.d. =  twice a day; NA =  not available; RYGB =  Roux-en-Y gastric bypass; SG =  sleeve gastrectomy; t.d.s. =  three times a day

^1^Adjusted to 600 mg t.d.s. in the pharmacokinetic calculations

^2^As needed

^3^Started before 1 month postoperatively

^4^Discontinued before 1 month postoperatively

^5^Started before 6 months postoperatively

^6^Started before 12 months postoperatively

Key pharmacokinetic data are presented in Table 2. Data for one gabapentin-treated patient were not available at 1 month, whereas data for one pregabalin-treated patient were not available at 12 months.

**Table 2:** Pharmacokinetic parameters preoperatively (baseline) and 1, 6 and 12 months postoperatively. In patients not using the same dose throughout the study, concentrations were adjusted to the main dose for that subject. All changes are displayed relative to preoperatively.

	Preoperatively (baseline)	One month postoperatively	Six months postoperatively	12 months postoperatively
**Gabapentin-treated patients (n = 2)**				
AUC, µmol/L**·** h	258 (247 to 269)	261^a^ –	234 (213 to 256)	271 (240 to 302)
AUC, % change	–	5.6^a^ –	−8.6 (−20.7 to 3.5)	5.6 (−10.9 to 22.2)
C_0_, µmol/L	24 (21 to 28)	22^a^ –	17 (12 to 23)	23 (22 to 25)
C_max_, µmol/L	42 (42 to 43)	39^a^ –	40 (40 to 41)	46 (43 to 49)
C_max_, % change	–	-7.1^a^ –	−4.7 (−7.0 to −2.4)	7.7 (0.0 to 15.5)
C_max_/C_0_ ratio	1.8 (1.5 to 2.0)	1.8^a^ –	2.6 (1.7 to 3.4)	1.9 (1.9 to 1.9)
t_max_, h	2.7 (2.5 to 3.0)	3.0^a^ –	2.0 (1.0 to 3.0)	1.0 (1.0 to 1.0)
t_1/2_, h	5.2 (5.2 to 5.3)	8.5^a^ –	6.6 (6.6 to 6.7)	6.4 (6.4 to 6.5)
CL/F, mL/min	173 (109 to 237)	224^a^ –	183 (137−229)	158 (122 to 194)
Vd/F, L	79 (49 to 109)	165^a^ –	106 (78 to 133)	88 (68 to 108)
**Pregabalin-treated patients (n = 3)**				
AUC, µmol/L**·** h	277 (239 to 327)	411 (319 to 525)	348 (279 to 391)	343^a^ (307 to 378)
AUC, % change	–	53 (19 to 120)	28 (5.3 to 64)	22^a^ (16 to 29)
C_0_, µmol/L	18 (15-24)	35 (19 to 57)	25 (20 to 30)	16^a^ (15 to 18)
C_max_, µmol/L	47 (39 to 52)	70 (58 to 84)	60 (45 to 67)	58^a^ (52 to 65)
C_max_, % change	–	55 (18 to 116)	31 (−8.2 to 71)	29^a^ (25 to 32)
C_max_/C_0_ ratio	2.7 (2.1 to 3.2)	2.4 (1.5 to 3.6)	2.5 (1.9 to 3.3)	3.6^a^ (2.9 to 4.3)
t_max_, h	1.5 (1.5 to 1.5)	1.0 (1.0 to 1.0)	1.2 (0.5 to 2.5)	1.0^a^ (1.0 to 1.0)
t_1/2_, h	8.7 (5.9 to 13.4)	8.0 (6.3 to 10.6)	8.1 (5.9 to 11.6)	7.5^a^ (5.9 to 9.1)
CL/F, mL/min	84 (66 to 96)	61 (30 to 80)	69 (40 to 84)	67^a^ (51 to 83)
Vd/F, L	59 (49 to 76)	39 (27 to 45)	44 (40 to 49)	41^a^ (40 to 42)

^a^Data not available for one patient

**Fig 1:**
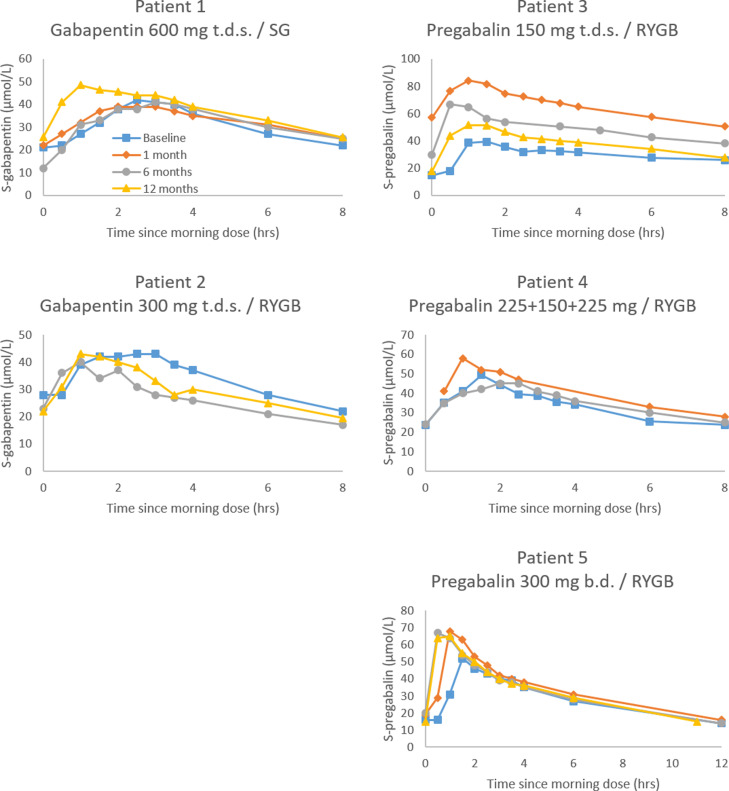
Individual time-concentration plots at baseline (preoperatively) and 1, 6 and 12 months postoperatively. In patients not using the same daily dose during the study, concentrations were adjusted to the main dose for that subject (Table 1). Note that the scale on the y-axis varies between subjects. RYGB =  Roux-en-Y gastric bypass; SG =  sleeve gastrectomy; b.d. =  twice a day; t.d.s. =  three times a day.

Individual concentration-time curves are presented in Fig 1.

**Fig 2:**
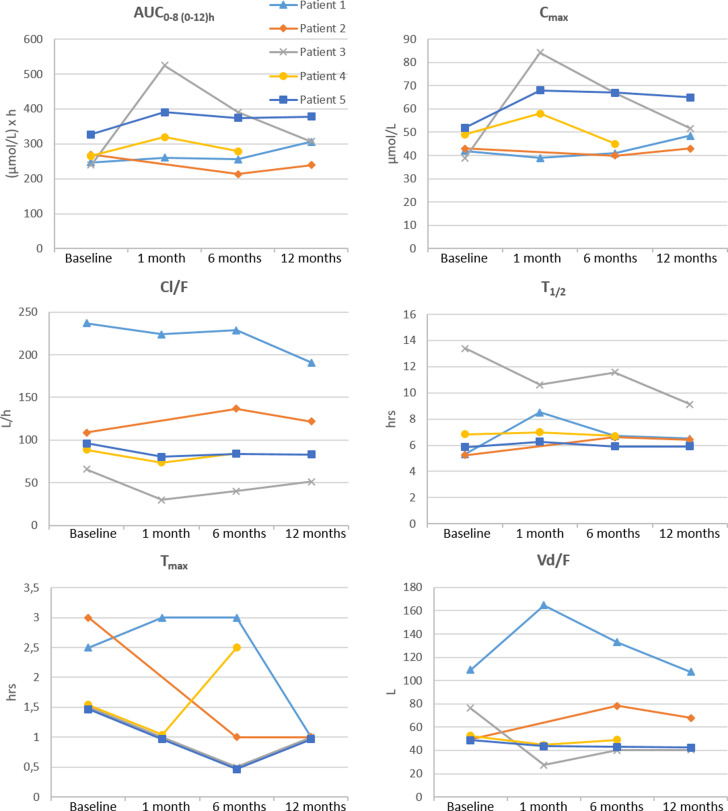
Pharmacokinetic parameters at baseline (preoperatively) and 1, 6 and 12 months after bariatric surgery in two patients treated with gabapentin (patients 1-2) and three patients treated with pregabalin (patients 3-5). Patient 1 underwent sleeve gastrectomy whereas the others underwent Roux-en-Y gastric bypass.

**Fig 3:**
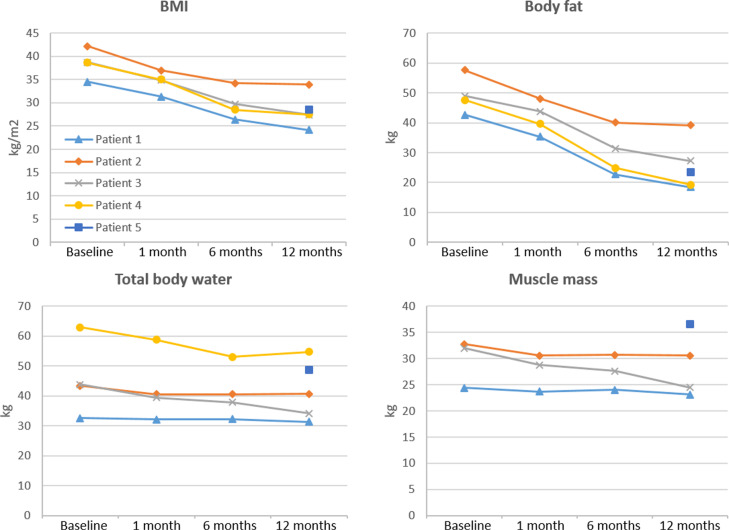
Body mass index (BMI), body fat, total body water and muscle mass at baseline (preoperatively) and 1, 6 and 12 months after bariatric surgery in two patients treated with gabapentin (patients 1-2) and three patients treated with pregabalin (patients 3-5). Patient 1 underwent sleeve gastrectomy whereas the others underwent Roux-en-Y gastric bypass.

Changes in pharmacokinetic variables and key body composition over the study period are depicted in Figs 2 and 3.

Body weight, BMI, body fat, muscle mass, visceral fat, and total body water values and changes throughout the study period are presented in S2 Table.

We observed the largest increase with regard to key pharmacokinetic parameters at one month postoperatively in pregabalin-treated patients, where we estimated a mean increase of 53% and 55% for AUC and C_max_, respectively, compared to baseline (Table 2). This increase was mainly due to large changes in one of the patients (patient 3), where we found AUC and C_max_ increases of 120% and 116%, respectively. This patient had also experienced the largest loss of muscle mass and total body water in the whole cohort at one month follow-up (Fig 3). At the same time, the decrease in V_d_/F was considerable (Fig 2). Serum concentrations, AUC and C_max_ subsequently decreased towards baseline (Figs 1 and 2). Of note, this patient also had consistently elevated C-reactive protein (CRP) values during the study period, which only normalized at the one-year follow-up. Unfortunately, eGFR was not available one month postoperatively but was otherwise relatively stable.

In the other two patients in the pregabalin group as well as the two patients in the gabapentin group, there were only minor changes in serum concentrations and pharmacokinetic variables throughout the observation period, although the decreases in total body mass, BMI and body fat were large.

## Discussion

One main finding in our study was the lack of substantial effects on gabapentin concentrations at various time points up to one year postoperatively. Likewise, Wallerstedt and associates reported unchanged gabapentin concentrations pre- vs. postoperatively, in two patients [15]. The minor impact of bariatric surgery on gabapentin concentrations may be related to its physicochemical and pharmacokinetic properties, being a relatively small and water-soluble molecule [15] that does not bind to plasma proteins [17] and is subject to renal excretion without any significant degree of metabolism [18].

Despite the physicochemical and pharmacokinetic similarities between gabapentin and pregabalin, we found larger effects of bariatric surgery in the pregabalin-treated patients. The largest extent of changes was observed one month postoperatively with a mean increase of 53% and 55% for AUC and C_max_, respectively, compared to baseline. However, this increase was mainly driven by changes in one 62-year-old female who had more than a two-fold increase in AUC and C_max_ at one month postoperatively (Fig 2). She was on a complex therapeutic regimen consisting of a total of nine medications in addition to pregabalin (Table 1). This patient experienced a loss of 10% of both muscle mass and total body water, which were the largest reported among all patients in our cohort during the first month postoperatively (Fig 3).

Interestingly, the only available previous study on the impact of bariatric surgery on pregabalin pharmacokinetics suggested an increase in dose-adjusted pregabalin trough concentrations, which was not observed for any other medication included in that study [15]. As the authors did not assess muscle mass or total body water changes, we cannot make direct comparisons with our findings. However, it is interesting that they reported about a 2.5-fold increase in median dose-adjusted pregabalin concentrations pre- vs. postoperatively in their patients [15], which is about the same increase as for AUC and C_max_ in our patient one month postoperatively.

Except for the larger decrease in body water and muscle mass, this patient did not clearly differ from the two other patients treated with pregabalin in any important variables recorded. It should be noted that the decrease in total body water was only about 10%, whereas the AUC more than doubled. Moreover, Cl/F was reduced with approx. 55% and V_d_/F dropped with about 60%. It therefore seems unlikely that alterations in body composition could be the sole explanation for the pharmacokinetic changes. Pregabalin is cleared by glomerular filtration [19] and obesity is associated with glomerular hyperfiltration [20]. Hence, decreased filtration with weight loss could, at least in theory, explain some of the concentration increase. However, there were no clear-cut alterations in the patient’s eGFR. Also, the elimination half-life did not change substantially. Another explanation could be a postoperative increase in the oral bioavailability, provided the patient had an unusually low bioavailability preoperatively. It could be speculated that transient local gastrointestinal postoperative changes could have led to a temporary increased bioavailability. Finally, we cannot exclude the possibility of imperfect medication adherence or dosing errors leading to the observed transient changes in one patient only.

Our study has several strengths. It is the first to provide full pharmacokinetic profiles at steady state conditions of gabapentin and pregabalin before and after bariatric surgery. Three measurement time points one, six and 12 months postoperatively enabled us to assess pharmacokinetic changes in the short- and longer-term. In addition, parallel to the changes in pharmacokinetic variables, we recorded alterations in body composition. On the other hand, there are also several limitations. First, a larger sample would be required to conclude whether bariatric surgery has the potential to influence gabapentinoid pharmacokinetics, and to better characterize the impact of specific types of surgery. Second, the lack of a control group losing weight using management options other than surgery [21] makes it difficult to know whether any pharmacokinetic changes are directly caused by the surgical procedure or are related to the weight loss and altered body composition. Third, the lack of surgical parameters, such as the length of bypassed bowel and the gastrojejunostomy size, hampered a better understanding of the pharmacokinetic impact of the bariatric surgeries. Fourth, given the lack of clinical outcomes, the clinical relevance of the pharmacokinetic changes is unclear.

In conclusion, based on our study the postoperative pharmacokinetic changes for gabapentin appear minimal, whereas for pregabalin there seems to be a possibility of increased concentrations shortly after surgery, at least in patients with large changes in body composition. Thus, therapeutic drug monitoring pre- and postoperatively could be considered to guide dosing.

## Supporting information

S1 Table Clinical biochemistry test results at baseline. Results for glomerular filtration rate and C-reactive protein are presented at baseline as well as at 1, 6 and 12 months postoperatively. Data are presented as mean (range).
(DOCX)

S2 Table Body composition variables preoperatively and at 1, 6 and 12 months postoperatively. Data are given as means with ranges in parentheses. All changes are displayed relative to preoperatively.
(DOCX)

S3 Table All collected data (Part I).
(PDF)

S4 Table All collected data (Part II).
(PDF)

S5 Table STROBE Checklist.
(DOCX)
