# Supplementary material for: Effects of sleeve gastrectomy and Roux-en-Y gastric bypass on the pharmacokinetics of gabapentin and pregabalin: A cohort study
Source: PLoS One. 2025 Mar 26;20(3):e0319912. doi: 10.1371/journal.pone.0319912 (PMC11940597; doi:10.1371/journal.pone.0319912)
Supplement: S1 Table — (DOCX) [file pone.0319912.s001.docx]

**S1 Table.** Clinical biochemistry test results at baseline. Results for glomerular filtration rate and C-reactive protein are presented at baseline as well as at 1, 6 and 12 months postoperatively. Data are presented as mean (range).

|  | **Gabapentin (n=2)** | **Pregabalin**  **(n=3)** |
| --- | --- | --- |
| Serum albumine (g/L) | 42 (39-44) | 43 (43-43) |
| Serum orosomucoid (g/L) | 0.90^a^ | 0.91^b^ |
| Serum ALAT (U/L) | 41 (15-67) | 34 (19-52) |
| Serum ASAT (U/L) | 33 (18-47) | 29 (20-36) |
| Serum gamma-GT (U/L) | 31 (15-47) | 38 (25-51)^a^ |
| Serum ALP (U/L) | 78 (41-115) | 85 (77-94) |
| Serum bilirubin, total (µmol/L) | 12 (8-15) | 8.7 (3-18) |
| Serum INR | 1.0 (0.9-1.1) | 0.9 (0.9-1.0) |
| eGFR (ml/min/1.73m^2^), preoperatively | 80 (67-94) | 94 (80-106) |
| eGFR (ml/min/1.73m^2^), 1 month postoperatively | 73^a^ | 111^b^ |
| eGFR (ml/min/1.73m^2^), 6 months postoperatively | 79 (69-88) | 99 (86-112) |
| eGFR (ml/min/1.73m^2^), 12 months postoperatively | 79^a^ | 105 (94-115)^a^ |
| Serum CRP, preoperatively (mg/L) | 5.7 (2.5-8.9) | 7.2 (2.5-11.0) |
| Serum CRP, 1 month postoperatively (mg/L) | 5.6 (2.5-8.7) | 4.8 (1.0-11.0) |
| Serum CRP, 6 months postoperatively (mg/L) | 2.0 (1.5-2.5) | 3.4 (1.0-7.8) |
| Serum CRP, 12 months postoperatively (mg/L) | 2.2 (1.9-2.5) | 1.8 (0.4-4.1) |

ALAT: alanine aminotransferase; ALP: Alkaline phosphatase; ASAT: aspartate aminotransferase; CRP: C-reactive protein, gamma-GT: gamma-glutamyltransferase; eGFR: estimated glomerular filtration rate; INR: international normalized ratio

^a^ Data missing for one patient

^b^ Data missing for two patients
