# Supplementary material for: Effects of sleeve gastrectomy and Roux-en-Y gastric bypass on the pharmacokinetics of gabapentin and pregabalin: A cohort study
Source: PLoS One. 2025 Mar 26;20(3):e0319912. doi: 10.1371/journal.pone.0319912 (PMC11940597; doi:10.1371/journal.pone.0319912)
Supplement: S2 Table — (DOCX) [file pone.0319912.s002.docx]

**S2 Table.** Body composition variables preoperatively and at 1, 6 and 12 months postoperatively. Data are given as means with ranges in parentheses. All changes are displayed relative to preoperatively.

|  | **Preoperatively**  **(baseline)** | **One month postoperatively** | **Six months postoperatively** | **12 months postoperatively** |
| --- | --- | --- | --- | --- |
| **Gabapentin (n=2)** | | | | |
| Body weight,  kg | 101.7  (87.1 to 116.4) | 91.2  (79.2 to 103.2) | 81.1  (66.8 to 95.4) | 77.9  (61.3 to 94.6) |
| Body weight,  % change^a^ | – | -10.2  (-11.3 to -9.1) | -20.7  (-23.3 to -18.0) | -24.2  (-29.6 to -18.7) |
| BMI,  kg/m^2^ | 38.3  (34.5 to 42.2) | 34.1  (31.3 to 37.0) | 30.3  (26.4 to 34.2) | 29.0  (24.2 to 33.9) |
| BMI,  % change | – | -10.8  (-12.3 to -9.3) | -21.2  (-23.5 to -19.0) | -24.8  (-29.9 to -19.7) |
| Body fat,  kg | 50.1  (42.6 to 57.6) | 41.7  (35.4 to 48.0) | 31.4  (22.7 to 40.1) | 28.8  (18.5 to 39.2) |
| Body fat,  % change^a^ | – | -16.8  (-16.9 to -16.7) | -38.5  (-46.7 to -30.9) | -44.2  (-56.6 to -31.9) |
| Muscle mass,  kg | 28.6  (24.4 to 32.8) | 27.1  (23.7 to 30.6) | 27.3  (24.0 to 30.7) | 26.8  (23.1 to 30.6) |
| Muscle mass,  % change^a^ | – | -4.8  (-6.7 to -2.9) | -4.0  (-6.4 to -1.6) | -6.0  (-6.7 to -5.3) |
| Visceral fat area, cm^2^ | 208  (167 to 249) | 193  (144 to 242) | 136  (70 to 202) | 116  (67 to 165) |
| Visceral fat area, % change^a^ | – | -8.5  (-13.5 to -2.8) | -38.5  (-57.9 to -19.0) | -46.8  (-60.0 to -33.6) |
| Total body water, kg | 38.0  (32.6 to 43.4) | 36.3  (32.1 to 40.6) | 36.4  (32.3 to 40.6) | 36.0  (31.4 to 40.7) |
| Total body water, % change^a^ | - | -4.0  (-6.4 to -1.5) | -3.7  (-6.4 to -0.9) | -4.9  (-6.2 to -3.7) |
| **Pregabalin (n=3)** | | | | |
| Body weight,  kg | 120.8^b^  (108.0 to 133.6) | 108.6  (97.4 to 119.9) | 90.1  (82.8 to 97.5) | 85.8  (73.6 to 94.1) |
| Body weight,  % change^a^ | - | -10.0^b^  (-10.2 to -9.8) | -25.2^b^  (-27.0 to -23.3) | -30.7^b^  (-31.8 to -29.6) |
| BMI,  kg/m^2^ | 38.6  (38.6 to 38.7) | 34.9  (34.9 to 35.0) | 29.1  (28.5 to 29.7) | 59.6  (34.2 to 89.7) |
| BMI,  % change | - | -9.6  (-9.8 to -9.3) | -24.7  (-26.2 to -23.2) | -29.0  (-29.2 to -28.7) |
| Body fat,  kg | 48.2  (47.6 to 48.9) | 41.7  (39.6 to 43.8) | 28.1  (24.9 to 31.4) | 23.3  (19.3 to 27.3) |
| Body fat,  % change^a^ | - | -13.6^b^  (-16.8 to -10.4) | -41.7^b^  (-47.7 to -35.8) | -51.8^b^  (-59.4 to -44.2) |
| Muscle mass,  kg | 32.0^c^ | 28.8^c^ | 27.6^c^ | 30.5^b^  (24.5 to 36.6) |
| Muscle mass,  % change^a^ | - | -10.0^c^ | -13.7^c^ | -23.4^c^ |
| Visceral fat area, cm^2^ | 178^c^ | 177^c^ | 127^c^ | 123^b^  (107 to 140) |
| Visceral fat area, % change^a^ | - | -0.9^c^ | -28.8^c^ | -40.1^c^ |
| Total body water, kg | 53.4^b^  (43.8 to 63.0) | 49.1^b^  (39.5 to 58.8) | 45.5^b^  (37.9 to 53.1) | 59.6  (34.2 to 89.7) |
| Total body water, % change^a^ | - | -8.2  (-9.8 to -6.7) | -14.6  (-15.7 to -13.5) | -17.5  (-21.9 to -13.0) |

BMI = Body mass index

^a^Changes relative to baseline

^b^Data missing for one patient

^c^Data missing for two patients
