# Supplementary material for: Effects of sleeve gastrectomy and Roux-en-Y gastric bypass on the pharmacokinetics of gabapentin and pregabalin: A cohort study
Source: PLoS One. 2025 Mar 26;20(3):e0319912. doi: 10.1371/journal.pone.0319912 (PMC11940597; doi:10.1371/journal.pone.0319912)
Supplement: S3 Table — (PDF) [file pone.0319912.s003.pdf]

Variable	Time	Patient 1	Patient 2	Patient 3	Patient 4	Patient 5
**Drug**		Gabapentin	Gabapentin	Pregabalin	Pregabalin	Pregabalin
**Adusted dose**		600 mg t.d.s.	300 mg t.d.s.	150 mg t.d.s.	225 + 150 + 225 mg	300 mg b.d.
**Age**		37	59	62	49	44
**Sex**		Female	Female	Female	Male	Male
**Type of surgery**		SG	RYGB	RYGB	RYGB	RYGB
**Other medications**		See Table 1	See Table 1	See Table 1	See Table 1	See Table 1
**Body composition variables**						
**Body weight (kg)**	Before surgery	87,1	116,4	108	133,6	
	1 month postoperatively	79,2	103,2	97,4	119,9	
	6 months postoperatively	66,8	95,4	82,8	97,5	
	12 months postoperatively	61,3	94,6	73,6	94,1	89,7
**BMI (kg/m²)**	Before surgery	34,5	42,2	38,7	38,6	
	1 month postoperatively	31,3	37	34,9	35	
	6 months postoperatively	26,4	34,2	29,7	28,5	
	12 months postoperatively	24,2	33,9	27,4	27,5	28,6
**Body fat (kg)**	Before surgery	42,6	57,6	48,9	47,6	
	1 month postoperatively	35,4	48	43,8	39,6	
	6 months postoperatively	22,7	40,1	31,4	24,9	
	12 months postoperatively	18,5	39,2	27,3	19,3	23,4
**Visceral fat area (cm²)**	Before surgery	166,7	249,3	178,2		
	1 month postoperatively	144,2	242,3	176,5		
	6 months postoperatively	70,1	202,0	126,8		
	12 months postoperatively	66,6	165,4	106,8		139,6
**Total body water (kg)**	Before surgery	32,6	43,4	43,8	63	
	1 month postoperatively	32,1	40,6	39,5	58,8	
	6 months postoperatively	32,3	40,6	37,9	53,1	
	12 months postoperatively	31,4	40,7	34,2	54,8	
**Muscle mass (kg)**	Before surgery	24,4	32,8	32		
	1 month postoperatively	23,7	30,6	28,8		
	6 months postoperatively	24	30,7	27,6		
	12 months postoperatively	23,1	30,6	24,5		36,6
**Pharmacokinetic variables**						
**$AUC_{0\text{-}Tlast}$ ((µmol/L)×h)**	Before surgery	247	269	239	265	327
	1 month postoperatively	261		525	319	390
	6 months postoperatively	256	213	391	279	375
	12 months postoperatively	302	240	307		378
**C_{max} (µmol/L)**	Before surgery	42	43	39	49	52
	1 month postoperatively	39		84	58	68
	6 months postoperatively	41	40	67	45	67
	12 months postoperatively	49	43	52		65
**C_0 (µmol/L)**	Before surgery	21	28	15	24	16

	1 month postoperatively	22		57	28	19
	6 months postoperatively	12	23	30	24	20
	12 months postoperatively	42	22	18		15
**Cl/F (L/h)**	Before surgery	237	109	66	89	96
	1 month postoperatively	224		30	74	80
	6 months postoperatively	229	137	40	84	84
	12 months postoperatively	194	122	51		83
**$T_{1/2}$ (h)**	Before surgery	5,3	5,2	13,4	6,8	5,9
	1 month postoperatively	8,5		10,6	7,0	6,3
	6 months postoperatively	6,7	6,6	11,6	6,7	5,9
	12 months postoperatively	6,5	6,4	9,1		5,9
**T_{max} (h)**	Before surgery	2,5	3	1,5	1,5	1,5
	1 month postoperatively	3		1	1	1
	6 months postoperatively	3	1	0,5	2,5	0,5
	12 months postoperatively	1	1	1		1
**Vd/F (L)**	Before surgery	109	49	76	53	49
	1 month postoperatively	165		27	45	44
	6 months postoperatively	133	78	40	49	43
	12 months postoperatively	108	68	40		42
**C_{max}/C_0**	Before surgery	2,00	1,54	2,67	2,08	3,25
	1 month postoperatively	1,77		1,47	2,07	3,58
	6 months postoperatively	3,42	1,74	2,23	1,88	3,35
	12 months postoperatively	1,90	1,95	2,90		4,33
**Clinical biochemistry variables**						
**Serum albumin (g/L)**	Before surgery	44	39	43	43	43
**Serum orosomucoid (g/L)**	Before surgery		0,90			0,91
**Serum ALAT (U/L)**	Before surgery	15	67	19	31	52
**Serum ASAT (U/L)**	Before surgery	18	47	20	31	36
**Serum GT (U/L)**	Before surgery	15	47	25		51
**Serum ALP (U/L)**	Before surgery	41	115	84	94	77
**Serum bilirubin (µmol/L)**	Before surgery	15	8	3	5	18
**Serum INR**	Before surgery	1,1	0,9	0,9	0,9	1,0
**Serum CRP (mg/L)**	Before surgery	2,5	8,9	11,0	8,0	2,5
	1 month postoperatively	2,5	8,7	11,0	1,0	2,4
	6 months postoperatively	2,5	1,5	7,8	1,0	1,4
	12 months postoperatively	2,5	1,9	4,1	1,0	0,4
**eGFR (mL/min/1.73m²)**	Before surgery	94	67	80	97	106
	1 month postoperatively		73			111
	6 months postoperatively	88	69	86		112
	12 months postoperatively		79	94		115
