# Supplementary material for: Effects of sleeve gastrectomy and Roux-en-Y gastric bypass on the pharmacokinetics of gabapentin and pregabalin: A cohort study
Source: PLoS One. 2025 Mar 26;20(3):e0319912. doi: 10.1371/journal.pone.0319912 (PMC11940597; doi:10.1371/journal.pone.0319912)
Supplement: S4 Table — (PDF) [file pone.0319912.s004.pdf]

Patient 1, gabapentin 600 mg t.d.s

(1200 mg t.d.s 12 months after surgery, concentrations given are adjsusted to 600 mg t.d.s)

Preoperatively		1 month after surgery		6 months after surgery		12 months after surgery	
Time after intake (h)	Concentration (µmol/L)	Time after intake (h)	Concentration (µmol/L)	Time after intake (h)	Concentration (µmol/L)	Time after intake (h)	Concentration (µmol/L)
0,0	21	0,0	22	0,0	12	0,0	25,5
0,5	22	0,5	27	0,5	20	0,5	41
1,0	27	1,0	32	1,0	31	1,0	48,5
1,5	32	1,5	37	1,5	33	1,5	46,5
2,0	38	2,0	39	2,0	38	2,0	45,5
2,5	42	2,5	39	2,5	38	2,5	44
3,0	41	3,0	39	3,0	41	3,0	44
3,5	40	3,5	37	3,5	40	3,5	42
4,0	36	4,0	35	4,0	38	4,0	39
6,0	27	6,0	31	6,0	30	6,0	33
8,0	22	8,0	25	8,0	25	8,0	25,5

Patient 2, gabapentin 300 mg t.d.s

Preoperatively		1 month after surgery		6 months after surgery		12 months after surgery	
Time after intake (h)	Concentration (µmol/L)	Time after intake (h)	Concentration (µmol/L)	Time after intake (h)	Concentration (µmol/L)	Time after intake (h)	Concentration (µmol/L)
0,0	28			0,0	23	0,0	22
0,5	28			0,5	36	0,5	31
1,0	39			1,0	40	1,0	43
1,5	42			1,5	34	1,5	42
2,0	42			2,0	37	2,0	40
2,5	43			2,5	31	2,5	38
3,0	43			3,0	28	3,0	33
3,5	39			3,5	27	3,5	28
4,0	37			4,0	26	4,0	30
6,0	28			6,0	21	6,0	25
8,0	22			8,0	17	8,0	19,5

Patient 3, gabapentin 150 mg t.d.s

Preoperatively		1 month after surgery		6 months after surgery		12 months after surgery	
Time after intake (h)	Concentration (µmol/L)	Time after intake (h)	Concentration (µmol/L)	Time after intake (h)	Concentration (µmol/L)	Time after intake (h)	Concentration (µmol/L)
0,0	14,6	0,0	57,1	0,0	29,9	0,0	17,8
0,5	18,0	0,5	76,7	0,5	66,8	0,5	43,9
1,0	38,5	1,0	84,2	1,0	64,8	1,0	51,6
1,5	39,3	1,5	81,6	1,5	56,2	1,5	51,4
2,0	35,7	2,0	74,7	2,0	53,8	2,0	46,5
2,5	31,8	2,5	72,5			2,5	42,5
3,0	33,2	3,0	70,0			3,0	41,3
3,5	32,3	3,5	67,8	3,5	50,5	3,5	39,9
4,0	31,6	4,0	65,0	4,6	47,9	4,0	38,8
6,0	27,6	6,0	57,6	6,0	42,6	6,0	34,2
8,0	25,9	8,0	50,5	8,0	38,0	8,0	27,7

Patient 4, preagabalin 225 + 150 + 225 mg/d

Preoperatively		1 month after surgery		6 months after surgery		12 months after surgery	
Time after intake (h)	Concentration (µmol/L)	Time after intake (h)	Concentration (µmol/L)	Time after intake (h)	Concentration (µmol/L)	Time after intake (h)	Concentration (µmol/L)
0,0	23,6	0,0		0,0	24,0		

0,5	35,3	0,5	41,0	0,5	35,0		
1,0	41,0	1,0	58,0	1,0	40,0		
1,5	49,3	1,5	52,0	1,5	42,0		
2,0	44,4	2,0	51,0	2,0	45,0		
2,5	39,5	2,5	47,0	2,5	45,0		
3,0	38,7	3,0		3,0	41,0		
3,5	35,6	3,5		3,5	39,0		
4,0	34,3	4,0		4,0	36,0		
6,0	25,6	6,0	33,0	6,0	30,0		
8,0	23,7	8,0	28,0	8,0	25,0		

Patient 5, pregabalin 300 mg b.d.

Preoperatively		1 month after surgery		6 months after surgery		12 months after surgery	
Time after intake (h)	Concentration (µmol/L)	Time after intake (h)	Concentration (µmol/L)	Time after intake (h)	Concentration (µmol/L)	Time after intake (h)	Concentration (µmol/L)
0,0	16,0	0,0	19,0	0,0	20,0	0,0	15,0
0,5	16,0	0,5	29,0	0,5	67,0	0,5	64,0
1,0	31,0	1,0	68,0	1,0	64,0	1,0	65,0
1,5	52,0	1,5	63,0	1,5	54,0	1,5	55,0
2,0	46,0	2,0	53,0	2,0	48,0	2,0	50,0
2,5	43,0	2,5	48,0	2,5	44,0	2,5	44,0
3,0	40,0	3,0	42,0	3,0	39,0	3,0	40,0
3,5	39,0	3,5	40,0	3,5	38,0	3,5	37,0
4,0	35,0	4,0	38,0	4,0	35,0	4,0	36,0
6,0	27,0	6,0	31,0	6,0	28,0	6,0	29,0
12,0	14,0	12,0	16,0	12,0	14,0	11,0	15,0
* Estimated 12-hour concentration, used in the caculations of AUC 0-12 and related variables						12,0	14,0*
